# Population Bottlenecks during the Infectious Cycle of the Lyme Disease Spirochete *Borrelia burgdorferi*

**DOI:** 10.1371/journal.pone.0101009

**Published:** 2014-06-30

**Authors:** Ryan O. M. Rego, Aaron Bestor, Jan Štefka, Patricia A. Rosa

**Affiliations:** 1 Laboratory of Zoonotic Pathogens, Rocky Mountain Laboratories, National Institute of Allergy and Infectious Diseases, National Institutes of Health, Hamilton, Montana, United States of America; 2 Faculty of Science, University of South Bohemia, České Budějovice, Czech Republic; 3 Institute of Parasitology, ASCR, Biology Centre, České Budějovice, Czech Republic; University of North Dakota School of Medicine and Health Sciences, United States of America

## Abstract

*Borrelia burgdorferi* is a zoonotic pathogen whose maintenance in nature depends upon an infectious cycle that alternates between a tick vector and mammalian hosts. Lyme disease in humans results from transmission of *B. burgdorferi* by the bite of an infected tick. The population dynamics of *B. burgdorferi* throughout its natural infectious cycle are not well understood. We addressed this topic by assessing the colonization, dissemination and persistence of *B. burgdorferi* within and between the disparate mammalian and tick environments. To follow bacterial populations during infection, we generated seven isogenic but distinguishable *B. burgdorferi* clones, each with a unique sequence tag. These tags resulted in no phenotypic changes relative to wild type organisms, yet permitted highly sensitive and specific detection of individual clones by PCR. We followed the composition of the spirochete population throughout an experimental infectious cycle that was initiated with a mixed inoculum of all clones. We observed heterogeneity in the spirochete population disseminating within mice at very early time points, but all clones displayed the ability to colonize most mouse tissues by 3 weeks of infection. The complexity of clones subsequently declined as murine infection persisted. Larval ticks typically acquired a reduced and variable number of clones relative to what was present in infected mice at the time of tick feeding, and maintained the same spirochete population through the molt to nymphs. However, only a random subset of infectious spirochetes was transmitted to naïve mice when these ticks next fed. Our results clearly demonstrate that the spirochete population experiences stochastic bottlenecks during both acquisition and transmission by the tick vector, as well as during persistent infection of its murine host. The experimental system that we have developed can be used to further explore the forces that shape the population of this vector-borne bacterial pathogen throughout its infectious cycle.

## Introduction

The dynamics of infection that occur when a microorganism colonizes a host and then persists under immune pressure can be followed with an experimental system that tracks the composition of the infecting population *in vivo*. The complexity of such studies is amplified when studying vector-borne pathogens that colonize and are transmitted between diverse environments of both invertebrate and vertebrate hosts [1]. Studies of bacterial pathogenesis tend to view the host as a static setting and, in the absence of other information, rely upon a simplified approach to follow the parameters of an infection. The availability of appropriate animal models, mathematical platforms and genetic tools permit a more comprehensive analysis, which can elucidate the dynamic interplays that occur during an infection [2].

*Borrelia burgdorferi* is the causative agent of Lyme disease, a vector borne zoonosis that is maintained in North America, Europe and Asia through a tick vector of the genus *Ixodes* and mammalian or avian hosts [3]–[11]. The *B. burgdorferi* transmission cycle begins when infected nymphal tick vectors, which have acquired *B. burgdorferi* at the larval stage, feed on naïve vertebrate hosts and transmit the spirochete. Uninfected larval ticks subsequently feed on these infected small mammals (reservoir hosts) and acquire the spirochete [5]. The maintenance of *B. burgdorferi* in both ticks and mammals depends upon differential expression of a number of genes at various stages of the infectious cycle in order to overcome physical, temporal and immune-mediated barriers [12]–[14]. Such barriers represent potential bottlenecks that can limit the infectious population and select for variants with increased fitness. These bottlenecks could contribute to the variable prevalence of particular *B. burgdorferi* strains or genospecies in geographically distinct endemic areas. Previous studies looking at variants within a *B. burgdorferi* population in an infectious setting have hypothesized that potential bottlenecks are both random [15]–[17] and mediated by selection [18], but the methods and techniques to demonstrate or distinguish between these possibilities were not available. More recent studies have provided insight into variation in the *B. burgdorferi* population, and barriers to colonization and dissemination encountered during infection of the murine host, but have not included analyses with the tick vector [19], [20].

The first studies to elucidate population dynamics during bacterial infections utilized tagged isogenic subpopulations, as conducted with *Yersinia pseudotuberculosis* [21] and subsequently used in conjunction with a mathematical model for *Salmonella* [22] and *Campylobacter* [23] species. Similarly, tagged isogenic subpopulations created in a pathogenic protist, *Trypanosoma brucei*, were followed during dissemination within an infected mammal, as well as during acquisition and transmission by the insect vector, the tsetse fly [1]. Very recently, work has been carried out with uropathogenic *E. coli* looking at population dynamics and distribution niches during acute and chronic urinary tract infection [24], [25]. These studies highlighted aspects of an infection that could not be discerned by looking at a homogenous infectious population, including stochastic selection of bacteria that colonize various organs in a mouse [22] and bottlenecks that allow the progression of random subpopulations of infectious organisms in the transmission and establishment of an infection [1], [26], [27].

To track and decipher the population dynamics and spatio-temporal distribution of *B. burgdorferi* within the tick vector and mammalian host, we generated a set of isogenic wild-type clones carrying unique sequence tags. The composition of clones in the infecting *B. burgdorferi* population was monitored across time, either within mice and ticks, or following transmission between them. Through this analysis we have identified parameters that influence the complexity of the *B. burgdorferi* population within an infected host, and significant bottlenecks that occur during acquisition and transmission of *B. burgdorferi*. All of these factors could shape the maintenance of distinct but equipotent *B. burgdorferi* strains in nature.

These data provide a solid foundation for future studies addressing the modeling of important parameters of *B. burgdorferi* infection that facilitate or limit the diversity of strains transmitted in nature. This is the first study to investigate the *in vivo* population dynamics of a vector-borne bacterial pathogen and provides a model for similar studies with other vector-borne pathogens. We will use this system in future studies to explore factors that shape the population structure of *B. burgdorferi* during transmission/dissemination, and assess how the acquired immune response of the host influences the dynamics of infection and transmission.

## Results

### Generation of isogenic infectious *B. burgdorferi* clones

**Figure 1:**
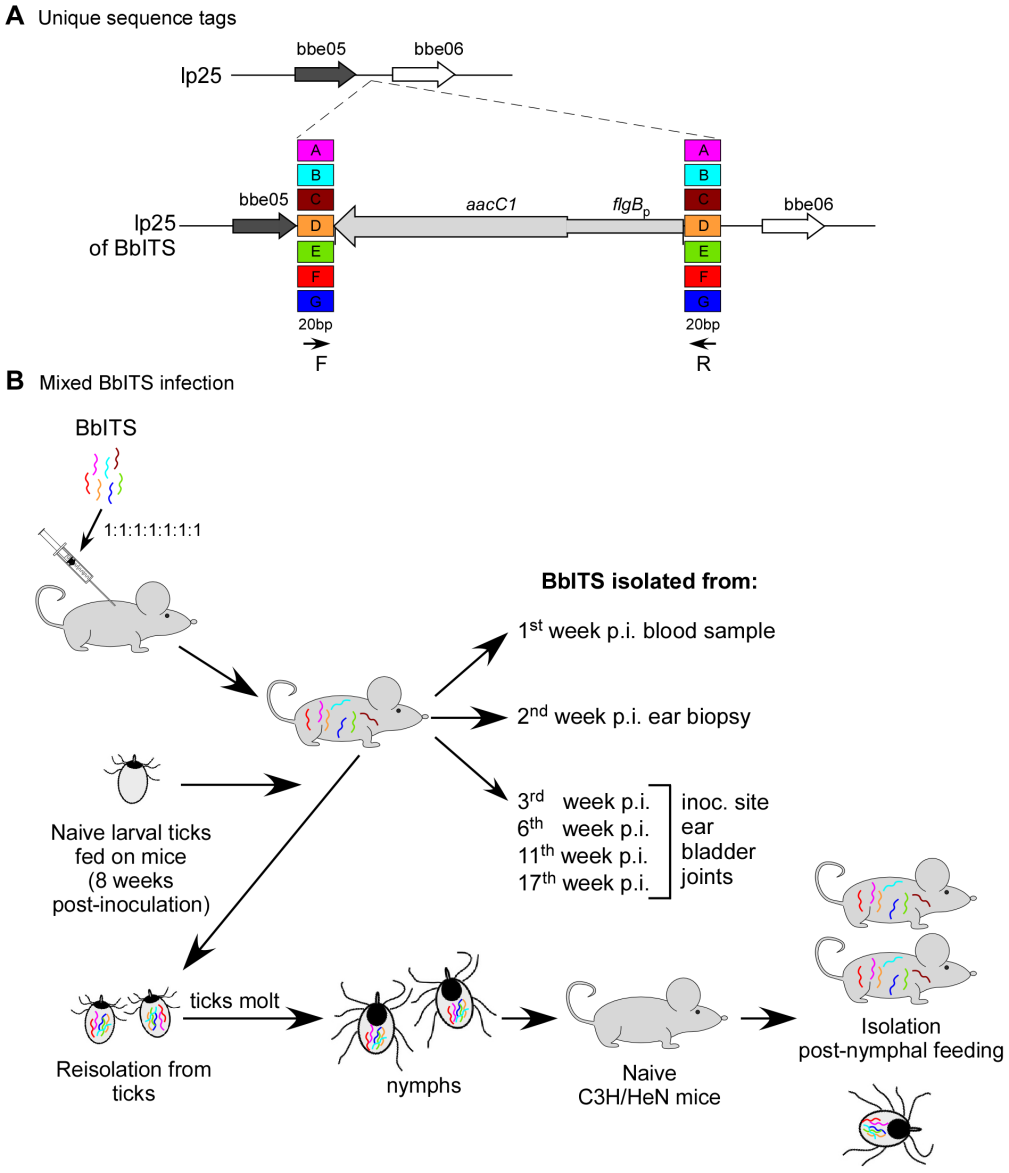
Experimental system to assess the population dynamics of *Borrelia burgdorferi* in mice and ticks. Seven isogenic wild type clones were constructed with unique 20*flgB*_p_::*aacC1* antibiotic resistance cassette (**A**). The individual clones were designated A to G on the basis of these unique tags, and collectively termed BbITS, for “*Borrelia burgdorferi* Isogenic Tagged Strains”. Tag-specific PCR primers (arrows “F” and “R”) direct the specific amplification of each clone. For the assessment of tagged strains in the context of a mixed infection, mice were injected with an inoculum containing equivalent numbers of all seven BbITS (**B**). Blood samples and ear punch biopsies were taken at 1 and 2 weeks post-inoculation, respectively, and cultured for spirochete isolation. Groups of mice were subsequently euthanized at various time points and spirochetes isolated from multiple tissues, as indicated. Infected mice were used to feed cohorts of approximately 100–200 larval ticks. Each infected tick cohort was maintained separately and ∼10 fed ticks from each cohort were individually cultured for spirochete isolation. The remaining BbITS-infected larval ticks were allowed to molt into nymphs and then fed on naïve mice, either individually or as groups of 5 ticks. Genomic DNA extracted from *B. burgdorferi* outgrowth cultures was used as template in PCR screens with tag-specific primers to identify the composition of the BbITS populations in larval and nymphal ticks, and the mice on which they had fed.

**Figure 2:**
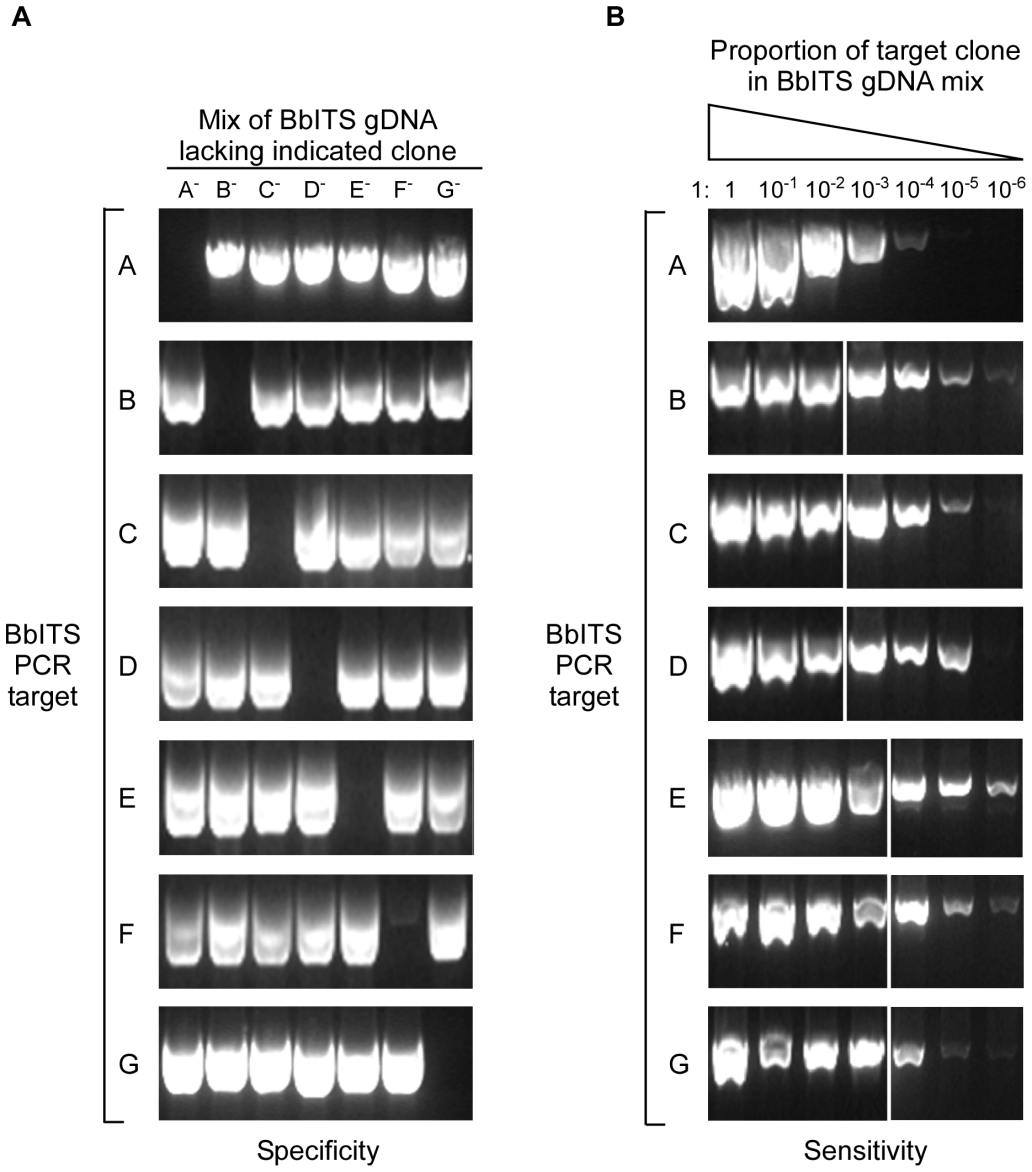
Sensitive and specific detection of individual BbITS with PCR primers for each unique sequence tag. The specificity of each PCR primer pair for detection of a single BbITS clone when amplified from a mixed DNA sample was demonstrated as shown in (**A**). Seven mixed templates were generated in which the genomic DNAs of 6 BbITS clones were combined in equal proportions, with a single clone omitted in succession, as indicated at the top of panel A. The amount of DNA used per PCR reaction was the equivalence of 2.4×10^7^ spirochetes total, or 4×10^6^ per clone. All 7 BbITS primer pairs, as identified on the left side of panel A, were tested with all 7 mixed samples. The sensitivity of each PCR primer pair for an individual BbITS clone when amplified from a mixed DNA sample was determined as shown in (**B**). 10-fold serial dilutions were prepared with genomic DNA for each individual BbITS clone, as indicated at the top of panel B. Seven mixed DNA templates were prepared from these dilution series with equal volumes of undiluted DNA from the remaining 6 clones. The dilution series spanned the range from all 7 BbITS clones present at the same amount (2.4×10^7^ total spirochetes), to an extreme of 1∶10^−6^ for the diluted clone relative to the other 6 BbITS. Each BbITS primer pair, as identified on the left side of panel B, was tested with the dilution series of its specific target DNA in the presence of undiluted DNA from the other 6 BbITS.

The population dynamics of *B. burgdorferi* in its vertebrate host and tick vector can be effectively probed with an experimental model system that tracks isogenic yet distinct spirochete subpopulations throughout the complete infectious cycle. To accomplish this, we first generated seven isogenic strains in the wild type clone B31-A3 background. Each clone carried unique 20 bp sequence tags flanking the *flgB*_p_::*aacC1* antibiotic resistance cassette inserted at a non-essential intergenic site on linear plasmid lp25 (Figure 1A). The seven clones were designated A to G on the basis of the unique tag they carried, and collectively termed BbITS, for “*Borrelia burgdorferi* Isogenic Tagged Strains”. Earlier work had shown that insertion of a selectable marker at this position on lp25 did not result in any phenotypic change relative to the wild type clone B31-A3 during the experimental mouse-tick infectious cycle [28]. The plasmid contents and *in vitro* growth rates of all seven clones were identical to each other and to B31-A3 (Figure S1 and data not shown). Likewise, all clones were equally infectious for mice by needle inoculation and efficiently acquired by larval ticks that fed on these infected mice (Table 1). PCR amplification with primers specific for each clone/tag amplified only the target DNA of that particular clone in a mixed sample of genomic DNA from all seven clones (Figure 2A). Additionally, each individual clone/tag could be detected by PCR amplification from a mixed sample of DNA even when the target sequence was substantially under-represented (dilution of 10^−4^ or less) relative to other clones (Figure 2B). Together these data demonstrate that all seven clones have comparable *in vivo* phenotypes and validate the specificity and sensitivity of a PCR-based approach to accurately track changes in the clonal composition of a mixed population of spirochetes represented by these BbITS.

**Table 1:** BbITS clones are infectious for mice and acquired by feeding larval ticks.

Strain^a^	# of mouse blood isolates/# of mice injected^b^^,^ c	# of seropositive mice/# of mice injected^c^^,^ d	# of isolates from larvae/# of ticks tested^e^
B31-A3	3/4	4/4	NA
BbITS-A	2/4	3/4	4/4
BbITS-B	4/4	4/4	6/8
BbITS-C	4/4	4/4	11/12
BbITS-D	2/4	2/4	3/7
BbITS-E	4/4	4/4	8/9
BbITS-F	3/4	3/4	12/12
BbITS-G	4/4	4/4	11/14

a 4 mice per wild-type or tagged strain were injected subcutaneously with 5×10^3^ spirochetes.

b A blood sample was obtained from each mouse at 1 week post-inoculation and spirochete isolation attempted from ∼150 µl of blood.

c Fisher's exact test comparisons indicated no significant differences between the number of mice infected by wild-type B31-A3 or by each BbITS.

d A blood sample was obtained from each mouse at 3 weeks post-inoculation and seroconversion assessed by immunoblot with *B. burgdorferi* whole cell lysates.

e Cohorts of 6–8 larval ticks were fed to repletion on 2 infected mice per strain at 4 weeks post-inoculation and spirochete isolation attempted from individual ticks 10 days after feeding. NA; not attempted.

### Mixed BbITS infection in mice

Having established a system with which to follow the population dynamics of *B. burgdorferi* infection, we next applied it to an experimental mouse-tick infectious cycle. Mixed infections were initiated in 36 mice by injecting a combined inoculum of 3.5×10^4^ BbITS (Figure 1B), with each tagged clone present at an infectious dose of 5×10^3^ spirochetes [29].

**Figure 3:**
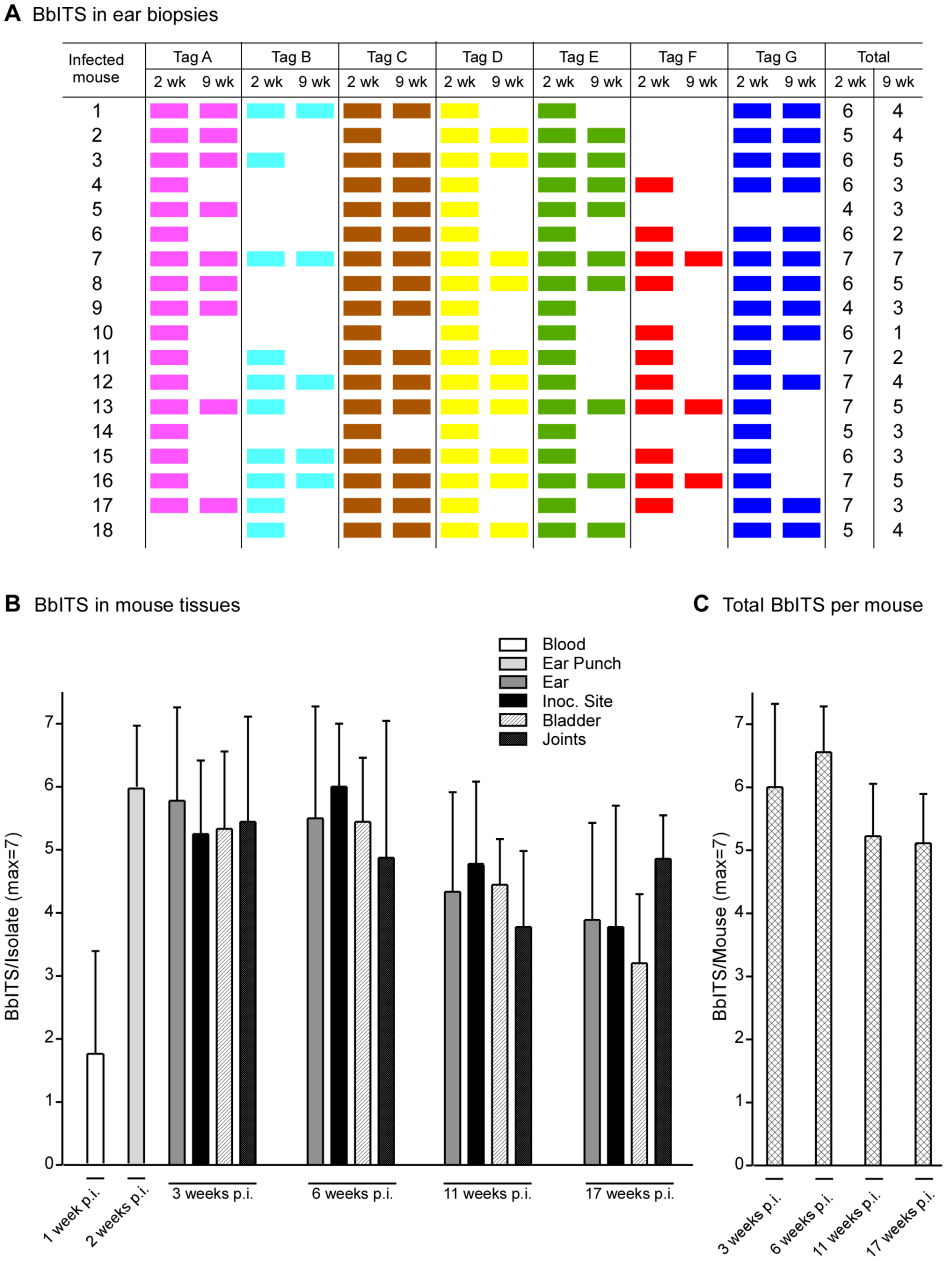
Decrease in complexity of *B. burgdorferi* population during persistent infection of mice. Mixed BbITS infections were initiated in 36 mice by injecting a combined inoculum of 3.5×10^4^ organisms, with each of 7 tagged clones (A to G) present at an infectious dose of 5×10^3^ spirochetes. Genomic DNA of *B. burgdorferi* from ear punch biopsies was used in PCR screens to identify the BbITS present in 18 individual mice at 2 and 9 weeks of infection, as shown in (**A**). Each tagged clone, identified at the head of each column, is represented by a different color, and the presence of a color block denotes detection of this clone in ear tissue at the indicated time. Groups of mice were subsequently euthanized at various times post-inoculation and spirochetes isolated from multiple tissues. Genomic DNA from isolates was used as template in PCR screens to identify the composition of the BbITS population in infected mouse tissues with time. These data are summarized in (**B**), where different tissues are identified in the key above the graph, and the time post-inoculation (p.i.) when spirochetes were isolated is identified below each set. The BbITS composition of the blood sample and ear punch biopsy was determined for all 36 mice, whereas BbITS in tissues at subsequent time points was determined with groups of 9 mice. The total number of BbITS recovered per mouse at each time point is shown in (**C**). Error bars show standard deviation from the mean. One way ANOVA was performed on the average number of BbITS present in a mouse with time of infection, giving P<0.01. Tukey's post hoc test of BbITS present at the 6^th^ and 11^th^ (or 17^th^) weeks of infection yielded P<0.01; similar *p* values were obtained when comparing the data from the 3^rd^ week with the 11^th^ or 17^th^ weeks of infection.

Isolates containing from 1 to 5 clones were recovered from blood samples of 25 mice one week after inoculation, whereas no isolates were obtained from the blood of the 11 other inoculated mice (Figure S2, Figure S3, Figure S4 and Figure S5). However, this early blood sample did not fully reflect the status or composition of disseminated *B. burgdorferi* infection, because isolates containing on average 6 clones were obtained from ear punch biopsies of all 36 mice at two weeks post-inoculation. The BbITS profiles in ear biopsies of 18 mice at both 2 and 9 weeks of infection are shown in Figure 3A. The number of *B. burgdorferi* clones/tags in ear biopsies of most mice decreased significantly with time (P<0.001), and no clones were isolated from the ears of any mice at 9 weeks that were not previously detected at 2 weeks. This indicates that the population of spirochetes that have disseminated to the ear by two weeks of infection is either representative of the entire BbITS population infecting a mouse, or that no spirochetes from other tissues re-colonize the ear after two weeks. These data also demonstrate the comparable proficiency of all 7 clones to establish infection in the competitive environment of a mixed infection, and the reliability and specificity of the PCR screen to document their presence or absence.

In addition to this analysis, the BbITS composition of spirochetes at the inoculation site and in the ear, bladder and joints were determined in groups of 9 mice euthanized at 3, 6, 11 and 17 weeks post-inoculation. These data are shown in Figures S2, S3, S4 and S5 documenting the BbITS composition in multiple tissues, with the earlier blood and ear biopsy results included for comparison. These data clearly demonstrate that the BbITS recovered from the blood a week after inoculation represent only a small subset of the *B. burgdorferi* clones that have established a disseminated infection in the mouse. This likely reflects the transient presence of *B. burgdorferi* in the blood and the relatively small sample of blood that was taken [30], [31]. In only one mouse was a clone detected in the blood at one week that was not also recovered from another tissue at three weeks (Figure S2, mouse #9, tag G). In general, the BbITS profile at 3 weeks of infection was similar for all tissues from the same mouse. These BbITS patterns held true later in infection, but there was a random reduction in clones recovered from the tissues of each mouse with time relative to what was detected in the ear biopsy of the mouse at the 2^nd^ week of infection (Figures S2, S3, S4 and S5).

Clearly, much can be learned from close scrutiny of the BbITS data from individual mice. However, as Figures S2, S3, S4 and S5 individually represent only a fraction of the data arising from this experiment, it illustrates the need to evaluate the results from a broader perspective in order to more fully appreciate the general trends during infection. To this end, a summary of all the data from the mixed BbITS infection of 36 mice by needle inoculation is shown in Figure 3B. This figure graphically depicts the average number of different *B. burgdorferi* clones recovered from each tissue, and collectively in each mouse, at sequential time points during infection. As mentioned above, spirochetes recovered from the blood at one week of infection inadequately represent the complexity of disseminated BbITS infection at subsequent time points. Blood and ear punch biopsies of all 36 mice in this experiment were analyzed at 1 and 2 weeks post-infection, respectively, whereas 9 mice per group were analyzed at subsequent time points. On average, each mouse was initially colonized by at least 6 of the 7 clones injected, as assessed by ear punch biopsy at two weeks of infection. The complexity of BbITS colonizing the skin and bladder remained fairly stable during the first six weeks of infection, and then gradually declined with time (P<0.01). The average number of clones in the joints did not follow this trend quite as closely, suggesting that the spirochete population in joints is subjected to somewhat lower pressures during persistent infection. We conclude that no strict bottlenecks exist during the initial dissemination and colonization of tissues by *B. burgdorferi* when infection of the mammalian host is experimentally initiated by subcutaneous injection of 3.5×10^4^ organisms comprising 7 distinct but equivalent clones.

### Acquisition of BbITS by naïve larval ticks feeding on mice with mixed infections

**Figure 4:**
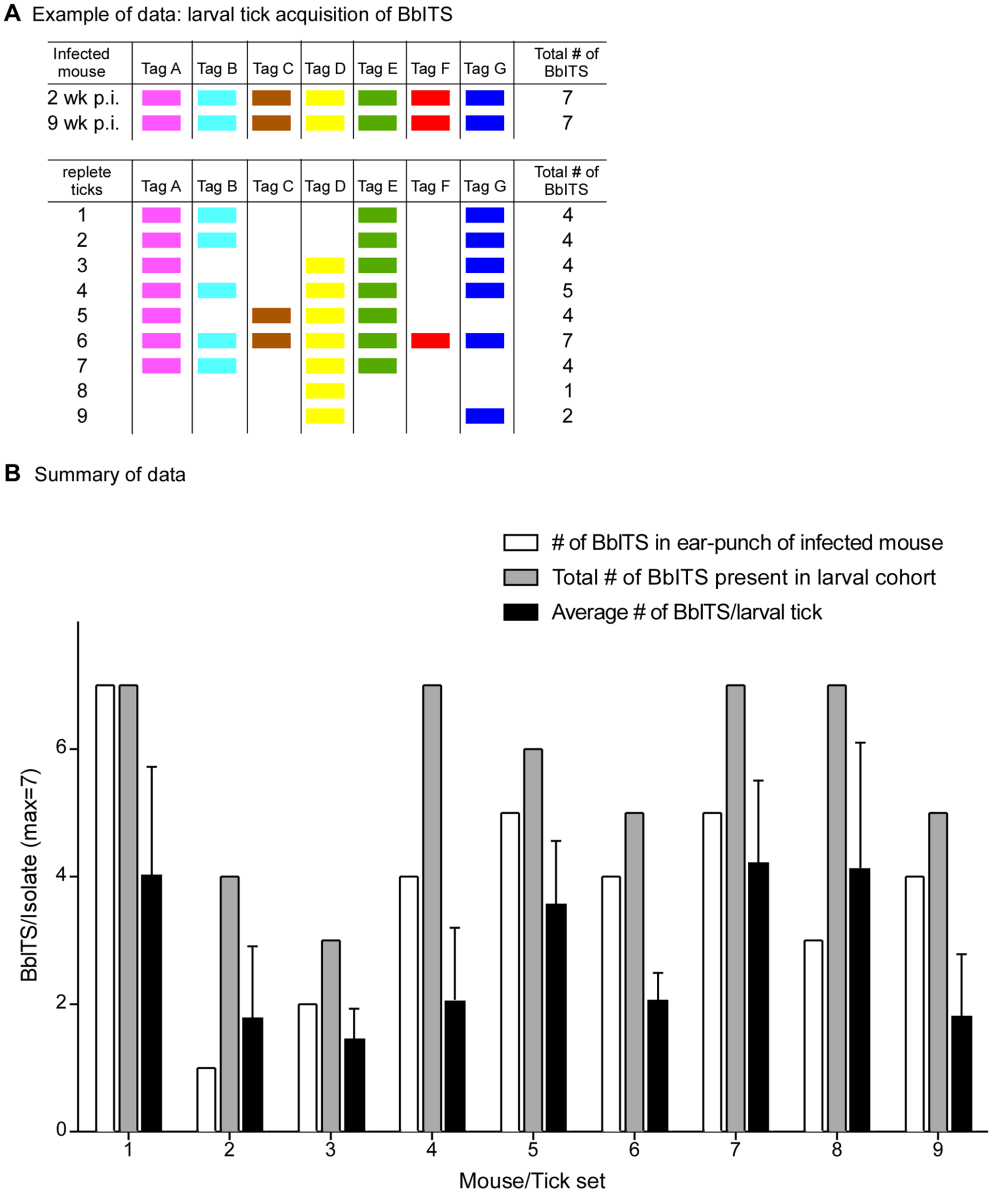
Bottleneck during acquisition of *B. burgdorferi* by fed larval ticks. Mixed BbITS infections were initiated by injecting mice with a combined inoculum of 3.5×10^4^ organisms, with each of 7 tagged clones (A to G) present at an infectious dose of 5×10^3^ spirochetes. Cohorts of larval ticks were fed on 9 infected mice at 8 weeks of infection and spirochetes isolated from ∼10 individual replete ticks per mouse. Genomic DNA of *B. burgdorferi* isolates was used in PCR screens to identify the BbITS present in both mice and ticks. An example of these data from a single infected mouse/fed tick cohort is shown in (**A**). Each tagged clone, identified at the head of each column, is represented by a different color, and the presence of a color block denotes detection of this clone in ear tissue at the indicated time (upper section), or acquired by individual replete ticks (lower section). These data pertain to mouse/tick set #1 of (**B**), in which the data for all 9 mouse/tick cohorts are summarized. Bars, identified in the key at the top of the graph, indicate the number of BbITS collectively present in each larval tick cohort, the average number of BbITS per individual tick in each cohort, and the total number of BbITS in the ear punch biopsy of each mouse on which these ticks fed. Error bars show standard deviation from the mean. A Mann-Whitney non-parametric test was used to compare the collective data for the total number of BbITS acquired by a larval tick cohort versus the number of BbITS present in an individual tick within the same larval cohort (P = 0.002).

We next asked whether acquisition and transmission of spirochetes during tick feeding represent potential bottlenecks during the natural infectious cycle. Acquisition was addressed by feeding naïve larval tick cohorts (∼100–200 larvae/mouse) on 9 mice initially infected with all seven BbITS clones, as determined by ear punch biopsy at 2 weeks post-inoculation. The larval tick feeding was done approximately 6 weeks later, at which point another ear biopsy was taken to determine the BbITS population persisting in each mouse at the time of tick feeding. Representative data from one of 9 mouse/tick sets are shown in Figure 4A, which depicts the BbITS present in the mouse at 2 and 9 weeks of infection, and the BbITS acquired by 9 individual ticks that fed to repletion on this same mouse. All 7 BbITS were present in the skin of this mouse at the time of tick feeding and collectively acquired by the cohort of fed larvae, but most individual ticks were colonized by only a subset of clones, no single clone was present in every fed tick, and only 1 tick acquired all 7 clones.

The BbITS profiles of all 9 mice and their corresponding fed tick cohorts are summarized in Figure 4B; mouse/tick set #1 represents the data presented in Figure 4A. The collective acquisition of BbITS from an infected mouse by a cohort of 10 larval ticks was quite efficient (mean number of BbITS in larval tick cohort  = 6) and often surpassed the number of BbITS detected in an ear punch biopsy from the same mouse at the time of tick feeding (mean number of BbITS detected in mouse  = 4). However, as previously illustrated in Figure 4A for mouse #1, most individual larvae acquired only a subset of clones present in the mouse on which they fed (mean number of BbITS acquired by an individual larval tick  = 3). This presumably reflects the relatively low spirochete burden in the skin of a persistently infected mouse and the limited sample size of either an ear punch biopsy or a single feeding tick. Together these data indicate that a bottleneck exists for acquisition of any particular *B. burgdorferi* clone by any given larval tick that feeds on a mouse with a mixed infection (P = 0.002), whereas the composition of clones acquired collectively by a cohort of ∼10 ticks accurately reflects the spirochete population in the infected mouse. The profile of BbITS in persistently infected mice and acquired by feeding larvae was random and comparable for all clones, demonstrating the equivalent status of all BbITS in mixed infections and stochastic clearance of clones during persistence (data not shown).

### Transmission of BbITS to naïve mice by infected nymphal tick cohorts

**Figure 5:**
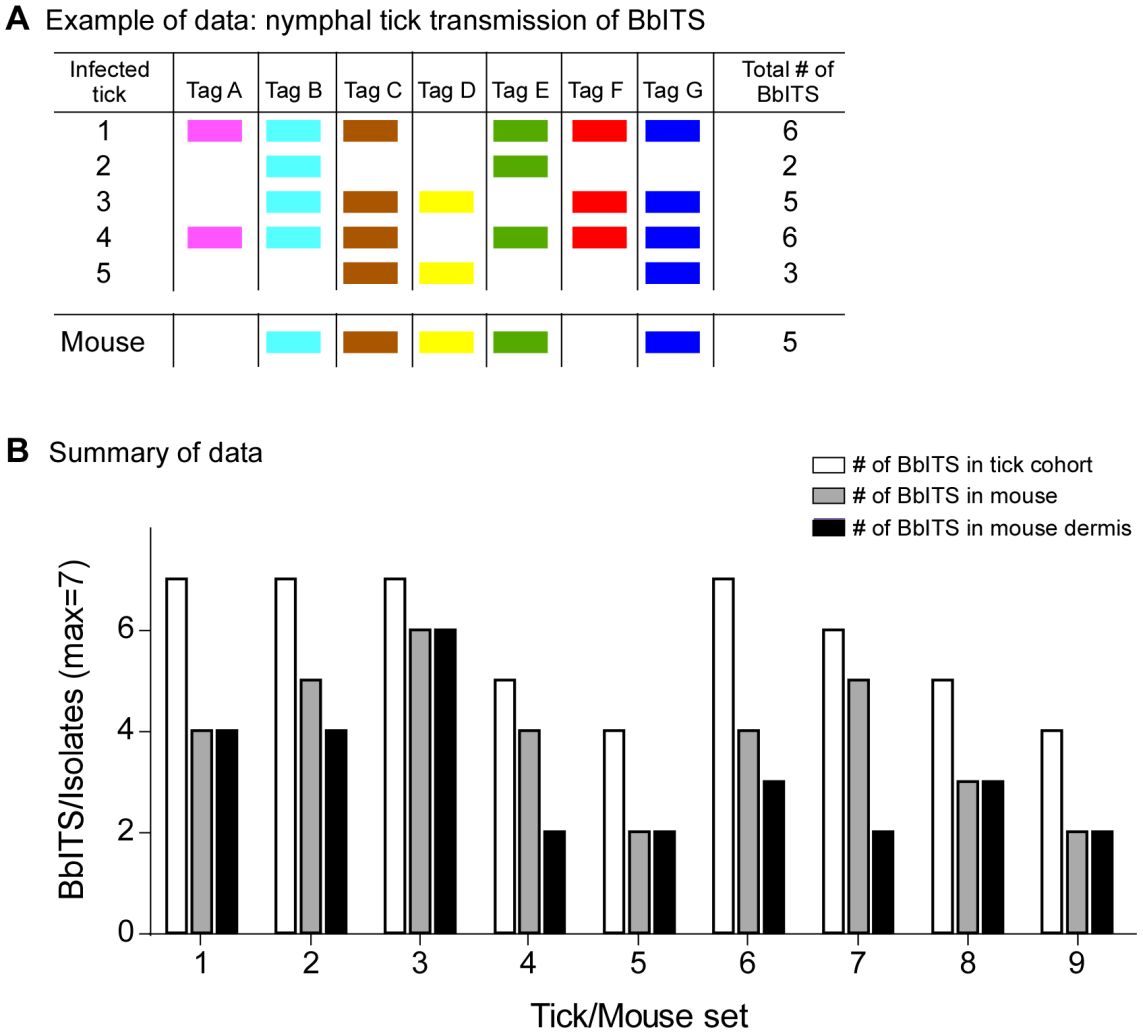
Bottleneck during transmission of *B. burgdorferi* from cohort of infected nymphal ticks. BbITS-infected nymphs were fed on 9 naïve mice (5 nymphs/mouse, from the same cohort of infected ticks analyzed as larvae in Figure 4), and spirochetes were isolated from individual replete ticks after drop-off and from mouse tissues approximately 5 weeks later. Genomic DNA of *B. burgdorferi* isolates was used in PCR screens to identify the BbITS population in both mice and ticks. An example of these data from one of the tick/mouse sets is shown in (**A**). Each tagged clone, identified at the head of each column, is represented by a different color, and the presence of a color block denotes isolation of this clone from a tick or from the mouse on which the cohort fed. These data pertain to tick/mouse set #2 of (**B**), in which the data for all 9 tick/mouse cohorts are summarized. Bars, identified in the key at the upper right of the graph, indicate total number of BbITS collectively present in the tick cohort, collectively present in isolates from 2 ears, 3 dermal sites, 2 joints, bladder, thymus and heart for each mouse, or collectively present in isolates from only dermal sites. A Mann-Whitney non-parametric test was used to compare the collective data for the total number of BbITS present in the nymphal tick cohort with the total number of BbITS present in the mouse, or the mouse dermis (P<0.0001 for both).

The population dynamics of *B. burgdorferi* during transmission to a naïve host were followed with the remaining BbITS-infected ticks after they had molted into nymphs. Transmission to 9 naïve mice was initially assessed with groups of 5 ticks per mouse. After feeding to repletion, the BbITS profiles of individual ticks and the resulting infections in mice were determined. Representative data for one of 9 mice and its cohort of fed ticks are shown in Figure 5A. Although all 7 BbITS were represented in the infected tick cohort, only 5 clones were recovered from the infected mouse. This does not reflect an inadequate sample size used for isolation because multiple tissues and organs, as well as ear samples from different times during infection, were utilized to determine the complete BbITS profile of each mouse.

The data for all 9 mice and their corresponding tick cohorts are summarized in Figure 5B. The trend suggested by the example of data shown in Figure 5A (tick/mouse set #2) was reinforced by all tick/mouse cohorts. The number of clones present in each infected mouse was fewer than the number of clones in the corresponding cohort of ∼5 ticks that fed on the same mouse. An average of 6 BbITS were represented in each tick cohort, whereas on average only 4 were recovered from each mouse (P<0.0001). This pattern is further reinforced when the BbITS composition of only the dermal sites is considered (P<0.0001). These data suggest that a bottleneck occurs during transmission or initiation of infection when *B. burgdorferi* is inoculated into the mammalian host by the natural route of tick bite. The constituent clones that successfully colonized each mouse were random, indicating a stochastic reduction in BbITS complexity with tick transmission (data not shown).

### Transmission of BbITS by individual nymphal ticks

In order to further investigate the potential bottleneck during tick-transmission of *B. burgdorferi*, nymphs colonized with mixed BbITS populations were individually fed on naive mice and the BbITS composition of individual fed ticks were subsequently determined. We also injected 2/3rds of each fed tick homogenate into a naïve mouse to compare the infectivity of their resident spirochete populations by tick bite versus needle inoculation. *B. burgdorferi* transmission was determined by isolation of spirochetes from various mouse tissues and identification of the BbITS present in infected mice.

**Figure 6:**
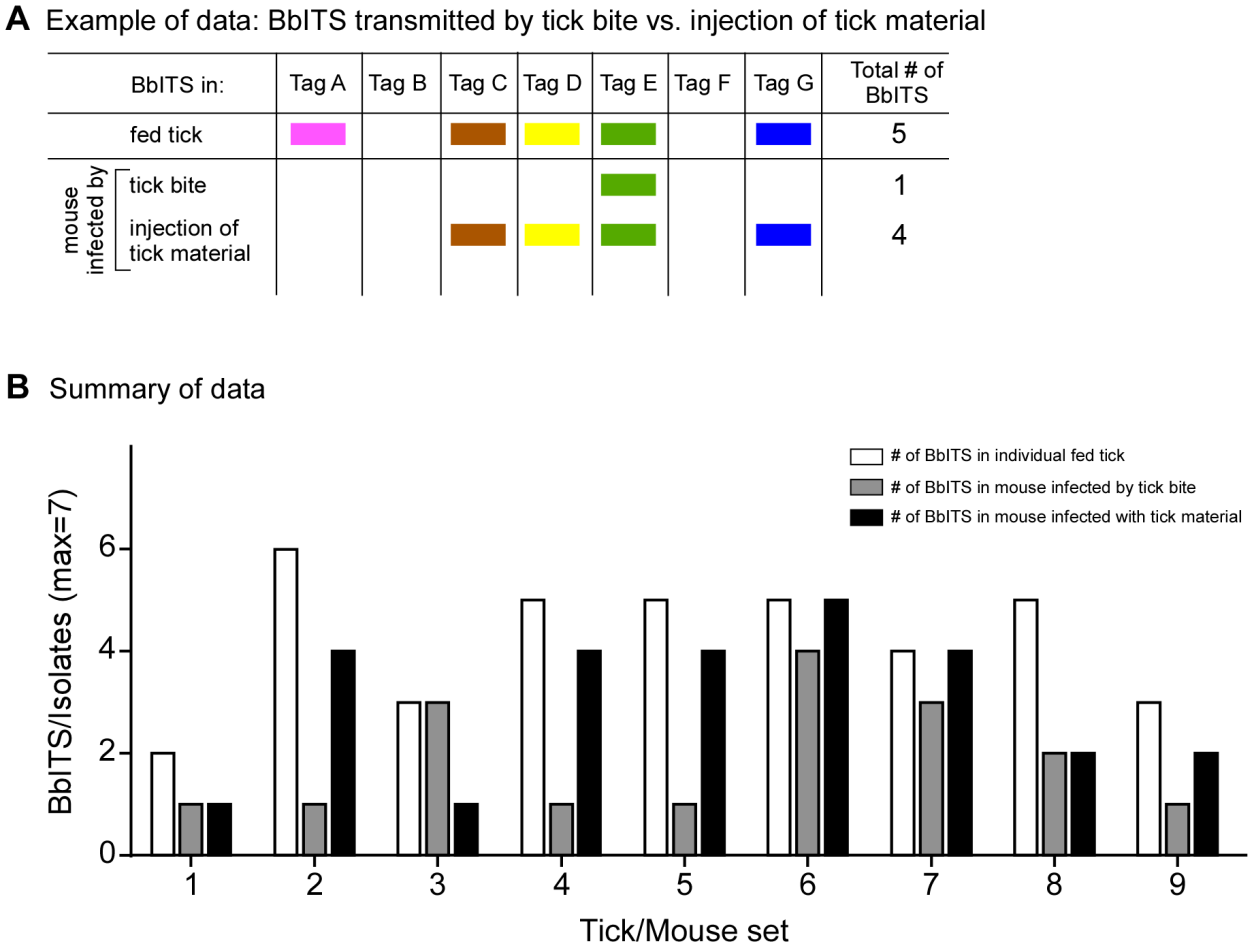
Inefficient transmission of BbITS by a single infected tick relative to injection of tick homogenate. Individual infected nymphs (from the same cohort of BbITS-infected ticks analyzed in Figures 4 and 5) were fed on naïve mice. Fed ticks were immediately crushed and a portion of each fed tick homogenate injected into another naïve mouse. Spirochetes were isolated from the remainder of the fed tick homogenate and from mouse tissues approximately 4 weeks later, from the same set of tissues as described in Figure 5. Genomic DNA of *B. burgdorferi* isolates was used in PCR screens to identify the BbITS population in both mice and ticks. An example of the data from a single tick/mouse infection is shown in (**A**). Each tagged clone, identified at the head of each column, is represented by a different color, and the presence of a color block denotes isolation of this clone from the fed tick or from the mice infected by either tick bite or injection of crushed tick material. The data for 9 individual tick/mouse sets are summarized in (**B**). Bars, identified in the key at the upper right of the graph, indicate total number of BbITS present in isolates from individual ticks or collectively present in tissues of the corresponding mice infected by either tick bite or by injection of fed tick homogenate. A Mann-Whitney non-parametric test was used to compare the collective data for the number of BbITS present in individual fed nymphal ticks with the number of BbITS present in the infected mice on which they had fed (P<0.001), or in the mice infected by injection of the same fed tick material (P = 0.834).

In general, the bite of a single infected tick (n = 23) was a relatively inefficient means of establishing mouse infection and succeeded less than half the time, whereas injection of a portion of the same fed tick material resulted in a mouse infection more than 80% of the time. This is consistent with our current understanding that only a small fraction of infectious *B. burgdorferi* colonizing the tick midgut are actually transmitted during tick feeding [32]–[34], and reflected in our assessment of the BbITS composition of individual ticks and the infected mice on which they fed. Representative data from one of 9 individual tick/mouse infections are shown in Figure 6A, and a summary of the data from all 9 tick/mouse infections is presented in Figure 6B. In every tick/mouse set examined, there was reduced complexity of the spirochete population (i.e., fewer BbITS) recovered from an infected mouse (mean number of BbITS  = 2) relative to what was present in the respective tick that fed upon it (mean number of BbITS  = 4; P<0.001), and typically a more “complete” infection could be established following injection of a mouse with the same tick-derived material (mean number of BbITS  = 3; P = 0.834) (Figure 6B). No bias was observed among the BbITS and the most abundant clones in an infected tick typically comprised the subset that was successfully transmitted (data not shown). Together, these data and the previous experiments demonstrate that tick transmission of *B. burgdorferi* imposes a bottleneck, which could influence the structure of the *B. burgdorferi* population in the natural infectious cycle. Whereas previous studies looking at the transmission of mixed infections (17,18,31) focused on adaptive mechanisms of the transmission, our data show that neutral processes may lead to similar outcomes.

### Transmission by co-feeding ticks infected with different BbITS clones

The previous experiments identified potential bottlenecks to transmission of *B. burgdorferi* from ticks with mixed BbITS infections. We also questioned the potential impact of co-feeding ticks infected with different strains of *B. burgdorferi* on transmission. In this experiment, 5 nymphal ticks from each of two cohorts individually infected with different BbITS as larvae (tagged clones D or F, same infected tick cohorts as described in Table 1) were allowed to simultaneously co-feed to repletion on individual mice (10 ticks collectively per mouse; 10 mice total). All 10 mice became infected and in most cases, both clones co-colonized all tissues analyzed (Table 2). This outcome indicates that the observed bottleneck in tick-transmitted infection with *B. burgdorferi* occurs within the tick or at the initial stage of mouse infection, and does not reflect competition among spirochetes for colonization sites following dissemination within the host. All of the ∼60 ticks analyzed after co-feeding remained uniquely infected with either clone D or clone F, indicating that acquisition of recently transmitted *B. burgdorferi* from the dermal feeding site by co-feeding nymphal ticks was not a frequent occurrence. These data also demonstrate that there is no negative impact on transmission of *B. burgdorferi* from one feeding tick by another infected tick co-feeding on the same host.

**Table 2:** Independent transmission of *B. burgdorferi* by co-feeding ticks.

	# of fed ticks recovered for each clone per mouse^a^		# of isolates for each clone/# of tissues cultured per mouse^b^	
Mouse No.	Clone D	Clone F	Clone D	Clone F
1	3	3	9/10	10/10
2	2	3	10/10	6/10
3	3	3	10/10	9/10
4	2	4	10/10	5/10
5	1	3	10/10	10/10
6	4	2	10/10	10/10
7	3	3	9/10	0/10
8	3	3	8/10	9/10
9	3	5	8/10	9/10
10	3	3	10/10	10/10

a 5 nymphal ticks from each of 2 cohorts infected individually with BbITS clone D or clone F were co-fed to repletion on each mouse. The number of ticks infected with each clone that were retrieved after feeding are as indicated. Genomic DNA of *B. burgdorferi* isolated from each crushed tick was used in PCR analysis to confirm the BbITS present.

b Detection of clone D or clone F by PCR screen of genomic DNA from *B. burgdorferi* isolates of both ears, 3 independent dermal sites, 2 joints, bladder, thymus and heart of each mouse.

## Discussion

Vector-borne zoonotic pathogens like *B. burgdorferi* encounter a number of diverse challenges that can shape their population as they cycle between the invertebrate vector and a vertebrate host. An unbiased experimental system represents a critical tool with which to investigate the dynamic structure of the bacterial population at different times in both settings. Natural populations of pathogens, including *B. burgdorferi*, contain a mixture of genetic strains with varying degrees of capability to infect hosts and disseminate to distal sites [35]–[39]. Such differences affect the course of infection in nature and in laboratory experiments that attempt to mimic such events. The current state of knowledge does not allow one to unambiguously estimate the cumulative effect of every individual gene mutation on the course of the transmission process. Hence, data collected using extensively large sample sizes are required to distinguish the effect of adaptive traits from that of neutral genetic drift. In this study we have followed a population of seven isogenic strains of *B. burgdorferi* throughout the spirochete's entire infectious cycle, encompassing dissemination, colonization and persistence in the mammalian host, acquisition by the tick vector, persistence through the molt to the nymphal stage and transmission by tick-bite to a naïve host. We were able to track this mixed *B. burgdorferi* population with a set of individually tagged, isogenic wild type clones (BbITS) (Figure 1). We utilized PCR, a simple and inexpensive technique, for highly sensitive and specific detection of each individual clone within the bacterial population throughout the infectious cycle (Figure 2). Although we did not pursue it in the current study, this PCR-based method may have the potential to be used in quantitating the relative proportion of individual BbITS in each population, as done in a previous study with *Salmonella* [22]. As noted by Troy and colleagues [19] in a study that used massive parallel sequencing to look at *Borrelia* subpopulations in mouse tissues, the only reliable way to detect minor spirochete types within the entire population, in mice or in ticks, is by expansion through outgrowth in media. Our investigation of the population dynamics of *B. burgdorferi* during a complete mouse-tick-mouse infectious cycle, in particular the tracking of spirochete sub-populations as they are acquired and then transmitted by feeding ticks, represents a distinctly different approach from those used in other recent reports [19], [20]. This has yielded significant and novel insights into the extent with which the (selectively neutral) drift shapes the spirochete population during transmission processes.

Utilizing this system to track the spirochete population in mice infected by needle inoculation, we observed efficient dissemination and colonization by all BbITS at the initial stage of murine infection, followed by a stochastic decrease in the number of individual clones present in different tissues at later time points (Figure 3). This reduction of population complexity coincides with a previously described decrease in the absolute number of spirochetes in tissues during chronic infection of the mammalian host [40], which is attributed to the ongoing pressure of the acquired immune system. This bottleneck should be taken into account when considering the likelihood of ticks acquiring *B. burgdorferi* during the larval blood meal. The reduction or disappearance of minority genotypes with increasing duration of the infection in a mammalian host, coupled with the length of time between the nymphal (transmitting) and larval (acquiring) blood meals, may affect the genetic composition of the pathogen population circulating in nature. Thus, in addition to ecological factors like the ability of hosts to migrate between demes or the prevalence of vectors in host populations, the effect of transmission bottlenecks due to drift should be taken into account as a factor influencing the complexity of the *B. burgdorferi* population within a region.

Throughout these experiments we did not observe any ‘re-seeding’ of clones from distal sites after host tissues were initially colonized, indicating that the transient blood-borne phase of spirochete dissemination that occurs early during infection of the mammalian host is not recurrent. This leads us to propose that in order to persist, a *B. burgdorferi* subpopulation needs to be established at a sufficient ‘threshold’ level in any given mouse tissue; otherwise this subpopulation will eventually be cleared as it wanes with time and cannot be supplemented by spirochetes from other sites. Assessing this *in vivo* scenario by q-PCR is problematic because it would require sampling the same site at different times from the same mouse, which is only feasible for dermal samples and would perturb the site and confound the outcome. The spirochete population in the joints, unlike other sampled tissues and organs, exhibited no obvious trend in population complexity that correlated with stage of infection (Figure 3). Liang and colleagues [40] showed that the joint was a protective niche, which allowed spirochetes to evade the acquired immune response during chronic murine infection. They suggested that the interaction between Decorin Binding Proteins (Dbp) on the surface of spirochetes and host decorin, which is more abundant in joints than in other tissues, could be providing this protection. Barthold and colleagues recently demonstrated that a *B. burgdorferi* mutant lacking Dbp had impaired survival and dissemination at the initial stage of infection in an immune-competent host [41], and that wild type spirochetes colonizing a decorin-rich region of the heart were less susceptible to immune-clearance than spirochetes in a decorin-poor region [42]. We believe that sub-populations of spirochetes present in the joint are somewhat impervious to the immune pressures that target spirochetes at other sites and thus not subjected to such strong bottleneck effects.

We next investigated the acquisition of *B. burgdorferi* by feeding larval ticks and found that a relatively small cohort of ticks (∼5) collectively acquired all spirochete subpopulations (BbITS) present in the infected mouse on which they had fed (Figure 4). However, individual replete ticks in the cohort acquired only a partial and random representation of the spirochete population, indicating a bottleneck at the point of larval tick feeding. Pertinent to this consideration, ingested spirochetes would presumably be exposed to the innate immune response of the tick [43]. *B. burgdorferi* has been shown to be resistant to some antimicrobial peptides [44], [45], but partial clearance of spirochetes shortly after tick acquisition could explain why all subpopulations present in the skin of an infected mouse do not effectively colonize a fed larval tick. However, artificial infection of larval ticks by a mixed culture of BbITS demonstrated efficient colonization of larval ticks by all clones to which they were simultaneously exposed (data not shown), suggesting that survival in the tick midgut is not a limiting event. Therefore, the probability of a spirochete being acquired by a larval tick likely reflects its abundance in the dermis at the feeding site.

In addition to the population bottleneck during larval tick acquisition, we observed further reduction in the complexity of the population following transmission of *B. burgdorferi* by nymphal ticks to a naïve mouse (Figures 5 and 6). Ohnishi and colleagues [33] demonstrated heterogeneity in the outer surface protein phenotype of wild type spirochetes present in the midgut and salivary glands of a feeding tick, or deposited into the skin of a mouse. Significantly, only a subset of these spirochetes was making OspC, an essential virulence factor for mouse infection by *B. burgdorferi* [46], [47]. The phenotypic heterogeneity of *B. burgdorferi* in a feeding tick likely contributes at least in part to the bottleneck in tick transmission that we observed in our current study with BbITS (Figures 5 and 6). We observed no difference in the number of clones present in the midguts and salivary glands of fed nymphal ticks (data not shown). In addition, we did not see any bottleneck when naïve mice were injected with crushed fed tick material (Figure 6), which undoubtedly represents a larger inoculum than is transmitted by tick bite. We suggest that most clones (BbITS) present in the tick midgut are transmitted by a feeding tick, but only those with a minimum threshold of viable spirochetes (expressing *ospC* and other RpoS-dependent genes) successfully colonize the host. Recent work has shown that the 50% infectious dose (ID_50_) by needle inoculation is approximately 100-fold lower for *B. burgdorferi* obtained directly from fed ticks [48], or from the mammalian host [49], relative to cultured organisms. Thus the *in vivo*-primed state of spirochetes, as well as components of tick saliva [50]–[53], contribute significantly to the dynamics of the *B. burgdorferi* population during early infection. A comparison of the data from mice infected with cultured organisms versus tick-transmitted spirochetes illustrates the contributions of both experimental models to an understanding of the spatio-temporal distribution of *B. burgdorferi* in the host.

In the current study we have analyzed the population dynamics of *B. burgdorferi* in naïve, but immune-competent, murine hosts. An important component of future BbITS studies will be experiments with immune-deficient mice to assess the contributions of the host's acquired immune response to the observed impacts on population complexity during chronic infection and larval tick acquisition. Similarly, we have monitored transmission of *B. burgdorferi* by nymphal ticks to immune-competent, but naïve, mice. A growing body of data indicates that OspC, while an essential virulence factor for *B. burgdorferi* infection of the mammalian host [46], [54], [55], also defines the serotype of the transmitted strain [35] and dictates the ability of tick-transmitted spirochetes to co-infect or super-infect an immune host [56], [57]. Isogenic but distinct BbITS will provide a powerful tool with which to test this hypothesis, as well as a means to evaluate the ability of OspC-based vaccines to elicit a broadly protective immune response. Such analyses should provide information pertinent to the maintenance of strain heterogeneity in endemic regions, as well as a measure of vaccine efficacy.

Models developed from our data may also help to explain the observed discrepancies between the distribution of spirochete genotypes found in reservoir hosts versus what is detected in tick vectors from the same endemic regions [58]–[61]. The prevalence of a particular *Borrelia* genotype in the skin of an infected host may dictate the probability of being acquired by a feeding tick, as well as its eventual clearance from the host. The bottleneck we identified during tick transmission may explain why not all genotypes detected in nymphal ticks can be identified in the reservoir hosts on which they feed. Analyses of the distribution of spirochete genotypes in reservoir hosts and ticks should encompass large datasets from broad geographic regions in order to identify biologically significant differences and eliminate stochastic variation.

A meta-analysis of *Borrelia* genospecies present in *Ixodes ricinus* ticks in Europe by Rauter and Hartung [62] did not identify a significant difference in the prevalence of mixed infection between nymphs and adult ticks, even though the latter would have taken an additional bloodmeal. These authors suggest that host complement in the midgut of the fed tick, ingested as part of the blood meal, could possibly explain this effect. The strictures on the spirochete population we have documented with this experimental model system should be taken into account in future studies of the dynamics of prevalence of *Borrelia* strains and genospecies in nature. Natural infestations of small rodents by tick larvae, nymphs, and adults are typically relatively limited, and usually only a small number of ticks feed simultaneously on an individual host [59], [63]. Seasonal fluctuations in prevalence and intensity of tick infestation [64], coupled with annual cycles of rodent population densities [65], [66], create conditions under which the frequency of host-vector interactions could be sufficiently restricted to allow drift to affect the composition of *Borrelia* populations maintained in the natural transmission process, in the same way as we have demonstrated in this experimental model.

We have identified several bottlenecks that stochastically limit the complexity of wild type *B. burgdorferi* during the mouse/tick infectious cycle. We now have an experimental system that can be used to model the population dynamics of *B. burgdorferi* during its natural infectious cycle. We anticipate that mathematical models based on these data can be used to predict key points when strategies to block transmission and reduce the prevalence of *B. burgdorferi* infection would be most effective.

## Materials and Methods

### Ethics Statement

The Rocky Mountain Laboratories, National Institute of Allergy and Infectious Diseases, National Institutes of Health, Animal Care and Use Committee (RML, NIAID, NIH, IACUC; USDA Permit Number: 51-F-0016 Customer #441, PHS number: A-4149-01) approved study protocols #2009-14, 2010-29 and 2011-55 for work conducted in strict accordance with the Guide for the Care and Use of Laboratory Animals of the National Institutes of Health. All infection studies were performed in an Animal Biosafety Level 2 (ABSL2) facility according to protocols reviewed and approved by the RML Institutional Biosafety Committee and the RML IACUC. All work in this study adhered to the institution's guidelines for animal husbandry, and followed the guidelines and basic principles of the Public Health Service Policy on Humane Care and Use of Laboratory Animals.

### *B. burgdorferi* clones and growth conditions

B31-A3 is an infectious clonal derivative [67] of the sequenced type strain B31 (ATCC 35210) that retains all the B31 plasmids except cp9 [68], [69]. Seven isogenic derivatives were constructed in the B31-A3 background, as described below. All strains were grown at 35°C in liquid Barbour Stoenner-Kelly II (BSKII) medium [70], [71] supplemented with 6% rabbit serum (Pel Freez Biologicals). Gentamicin (40 µg ml^−1^) was added when appropriate.

### Allelic exchange constructs

A previously described allelic exchange construct, p25, was modified to target unique sequence tags to a non-essential site on lp25 [28]. Seven different PCR primer sets with unique 20 bp sequences at both ends (Table S1) were used to amplify the *flgB*p*::aac*C1 cassette, which confers gentamicin resistance in *B. burgdorferi* and *E. coli* [72]. The amplified fragments were cloned into PCR 2.1 TOPO plasmid (Life Technologies), resulting in pTAG-(A to G) for the seven unique sequence tags. Each pTAG plasmid was digested with *BstZ17*I and *Sca*I, and ligated with *Sma*I-digested p25 using T4 DNA ligase (New England Biolabs). The ligations were then transformed into *E. coli* Top10 cells (Life Technologies) and transformants were selected on LB plates containing gentamicin. Primers in flanking *bbe05* and *bbe06* sequences (Table S1) were used to identify clones containing the tagged inserts, which were confirmed by sequencing, yielding allelic exchange constructs p25TAG-(A to G).

### Transformation of *B. burgdorferi* and generation of BbITS

20 µg of plasmid DNA purified from *E. coli* for each p25TAG-(A to G) was electroporated into *B. burgdorferi* clone B31-A3 as described previously [67], [73]. Electroporated cells were resuspended in 5 ml of BSKII medium and allowed to recover for approximately 24 hours at 35°C. Aliquots of the transformation mix were then plated in solid BSK medium supplemented with gentamicin at 40 µg ml^−1^. Colonies arising within 2 weeks after plating were screened by PCR using primers Gent-F and Gent-R (Table S1). Six positive colonies from each p25TAG transformation were aspirated into 10 ml of BSK II liquid medium and grown to mid-log phase. Glycerol stocks were made with 3 ml of the culture, while the remaining 7 ml was used to prepare total genomic DNA using Wizard genomic DNA kit (Promega). To ensure isogenicity with the parental B31-A3 strain, the total plasmid content of each transformant was determined by PCR screening using primers specific for each plasmid [67]. One isogenic clone for each of the seven tags on lp25 was selected for subsequent experiments. These tagged isogenic clones were termed “BbITS” for “*Borrelia burgdorferi* Isogenic Tagged Strains” (Figure 1A).

### *In vitro* growth rate determination

B31-A3 and the 7 individual BbITS were grown from frozen glycerol stocks to mid-log phase density of approximately 5×10^7^ spirochetes ml^−1^. Each clone was then inoculated in triplicate into fresh medium at an initial density of 1×10^5^ spirochetes ml^−1^. Spirochete density was enumerated every 24 hours for a period of 72 hours using a Petroff-Hausser chamber and dark-field microscopy (Figure S1).

### Animal Infections

The Rocky Mountain Laboratories (RML) are accredited by the International Association of Assessment and Accreditation of Laboratory Animal Care. Animal protocols were prepared according to the guidelines of the National Institutes of Health and approved by the RML Animal Care and Use Committee. For assessing the infectivity of individual BbITS in mice and their acquisition by larval ticks, C3H/HeN mice (Harlan Sprague-Dawley) were used and 4 mice per tag were injected subcutaneously with 5×10^3^ spirochetes. A blood sample was obtained at 1 week post-inoculation and 150 µl of blood was put into 10 ml of BSK-H complete (Sigma) with *Borrelia* antibiotics (20 µg/ml phosphomycin, 50 µg/ml rifampicin, 2.5 µg/ml amphotericin) [74], followed a week later by transfer of 7 ml of culture into 3 ml of fresh BSKII media. 2 mm ear punch biopsies were taken at 2 weeks post-inoculation and cultured in 10 ml of BSKII containing *Borrelia* antibiotics. At 3 weeks post-inoculation, blood samples were obtained. Larval *I. scapularis* ticks were allowed to feed on mice that were seropositive. These mice were then euthanized by an overdose of isoflurane followed by cervical dislocation. Spirochetes were isolated from ear tissues, bladders and rear-ankle joints [28], [55].

To assess tagged strains in the context of a mixed infection, 36 mice were injected with all seven BbITS (5×10^3^ spirochetes of each clone), at a combined inoculum of 3.5×10^4^ spirochetes (Figure 1B). As before, blood samples were taken at one week post-inoculation and 2 mm ear punch biopsies were obtained at 2 weeks post-inoculation and cultured for spirochete isolation. Blood samples were also taken at 3 weeks post-inoculation and used to assess seroconversion to *B. burgdorferi* proteins. Mice were euthanized as before, in groups of 9 animals, at 3, 6, 11 and 17 weeks post-inoculation and spirochetes were isolated from the ear, rear ankle joints, inoculation site, heart and bladder. Genomic DNA was extracted from *B. burgdorferi* isolates and used as template in PCR screens (described below) to identify the composition of the BbITS population in infected mice.

### Experimental mouse-tick cycle

#### Acquisition by larval ticks

Two seropositive mice per tagged clone from the individual BbITS infection study were used to feed cohorts of approximately 100 larval ticks per mouse to repletion. 6–8 ticks/mouse were individually crushed 10 days post-feeding in 1 ml of BSKII and plated to determine the spirochete load and infectivity of each tick cohort for each BbITS clone. For mixed infections, mice (n = 9) injected with equal proportions of all seven BbITS and infected with all 7 clones at 2 weeks post-inoculation, (as determined by ear punch biopsy) were used to feed cohorts of approximately 100–200 larval ticks. This tick feeding was done between 8 and 9 weeks post-inoculation, at which time another ear punch biopsy was taken for isolation of spirochetes and determination of the BbITS present in infected mice at the time of tick feeding. Each tick cohort was then maintained separately post-feeding. Approximately 10 fed larval ticks from each of the nine co-infected mice were individually crushed in 1 ml of BSKII and then transferred into 9 ml of BSKII with *Borrelia* antibiotics for isolation of spirochetes. Genomic DNA extracted from *B. burgdorferi* outgrowth cultures was used as template in PCR screens to identify the composition of the BbITS populations in larval ticks and the mice on which they had fed.

#### Transmission by cohort of infected nymphs

The remaining BbITS-infected larval ticks were allowed to molt into nymphs and then fed on an outbred colony of Swiss-Webster mice maintained at RML. For group feedings, 5 nymphs were fed per mouse. Ticks were individually crushed in 1 ml of BSKII at 2–3 days after drop-off and the homogenate transferred to 9 ml of liquid BSKII medium containing *Borrelia* antibiotics. Genomic DNA prepared from outgrowth cultures was screened by PCR to determine the composition of the BbITS population colonizing individual nymphal ticks. Ear punch biopsies were taken from mice 2 weeks after nymphal tick feeding to determine the BbITS population that had been transmitted and disseminated within the mouse at this time-point. Mice were bled at 3 weeks post-nymphal tick feeding, and seroconversion assessed. Mice were then euthanized and the following tissues and organs were placed in 10 ml of liquid BSKII with *Borrelia* antibiotics for reisolation of spirochetes: ears, skin biopsies from the upper back and both flanks, bladder, rear ankle joints, thymus and heart. Genomic DNA was extracted from resulting isolate cultures and screened by PCR to determine the BbITS population within each tissue or organ.

#### Transmission by individual infected nymphs

Individual infected nymphs were allowed to attach and feed on naïve mice (1 tick per mouse) to assess BbITS transmission from a single tick. Fed ticks were immediately crushed and the homogenates were used to determine the number of spirochetes in each tick by colony formation in solid medium (data not shown), and the BbITS composition of each tick by PCR screening of the outgrowth in liquid medium. In some cases, a portion of the fed tick homogenate was also injected into a naïve mouse for comparison of BbITS transmission by tick challenge versus needle inoculation. 3 weeks after challenge by tick feeding or injection, mice were bled and tested for seroconversion. All mice were then euthanized and spirochetes isolated from the same set of tissues and organs described above. Genomic DNA from isolates was screened by PCR to determine the BbITS population present in infected mouse tissues.

#### Transmission by co-feeding nymphs infected with different clones

For co-feeding experiments, 5 ticks from two different individual BbITS cohorts were allowed to feed to repletion on individual mice. The fed ticks were recovered and crushed in 200 µl of BSKII. These tick homogenates were used to determine the number of spirochetes by colony formation in solid medium (data not shown), and the BbITS composition by PCR screening of the outgrowth in liquid medium. 3 weeks after challenge by tick feeding, mice were bled and tested for seroconversion as before. All mice were then euthanized and spirochetes isolated from the same set of tissues and organs described above. Genomic DNA from isolates was screened by PCR to determine the BbITS population present in infected mouse tissues.

### PCR detection of BbITS in mouse and tick isolates

Genomic DNA was extracted from *B. burgdorferi* directly cultured from infected mouse tissues or crushed ticks (8 ml at 10^8^/ml) using the Wizard genomic DNA kit (Promega) per the manufacturer's instructions. Genomic DNA was eluted in a final volume of 100 microliters and 3 microliters (equivalence of 2.4×10^7^ spirochetes) was used per PCR reaction to determine BbITS composition. Seven PCR primer pairs specific for the unique tag in each BbITS clone (Table S1) were used individually with HotStarTaq DNA Polymerase (Qiagen) and the manufacturer's reagents and recommended reaction conditions. The following PCR parameters were used: an initial denaturation at 95°C for 5 min, followed by 30 cycles of 95°C for 45 seconds, 55°C for 45 seconds, 72°C for 1 minute, and a final extension at 72°C for 10 minutes.

The specificity of each primer pair for detection of a single BbITS clone when amplified from a DNA sample containing a mixture of clones was demonstrated as follows. Genomic DNA was extracted from individual cultures of all 7 BbITS clones and used to generate a series of mixed templates in which the DNAs of 6 BbITS clones were combined in equal proportions, with a single clone omitted in succession. The amount of DNA used per PCR reaction was comparable to that described above (equivalence of 2.4×10^7^ spirochetes). All 7 BbITS primer pairs (Table S1) were tested with all 7 samples using the PCR conditions described above (Figure 2A).

The sensitivity of each primer pair for an individual BbITS clone when amplified from a mixed DNA sample was determined as follows. A 10-fold serial dilution series was prepared with genomic DNA for each individual BbITS clone, extracted as described above. DNA template mixes were prepared from these dilution series with equal volumes of undiluted DNA from the remaining 6 clones. The dilution series spanned the range from all 7 BbITS clones present at the same amount (equivalence of 2.4×10^7^ spirochetes), to an extreme of 1∶10^−6^ for the diluted clone relative to the other 6 BbITS. Each BbITS primer pair (Table S1) was tested with the dilution series of its target DNA in the presence of undiluted DNA from the other 6 BbITS (Figure 2B).

### Statistical analyses

Statistical analyses were performed using the GraphPad program (GraphPad Software Inc.). P values were calculated using a two-tailed Mann-Whitney non-parametric comparison or One-way Analysis of Variance (ANOVA), followed by Tukey's post hoc test when multiple comparisons were made. Fisher's Exact Test was used to compare mouse infection data for individual BbITS with wild type. P values have been stated only for comparisons that were statistically significant.

## Supporting Information

Figure S1 **Identical** ***in vitro*** **growth phenotypes of BbITS and wild type** ***B. burgdorferi*** **.** Wild-type clone B31-A3 and the 7 individual BbITS were grown to mid-log phase density of approximately 5×10^7^ spirochetes ml^−1^. Each clone was then inoculated in triplicate into fresh medium at an initial density of 1×10^5^ spirochetes ml^−1^. Spirochete density of each culture was enumerated every 24 hours for a period of 72 hours using a Petroff-Hausser chamber and dark-field microscopy. Each strain is represented by a different color and symbol, as identified in the key, and error bars indicate standard deviation from the mean.
(TIF)

Figure S2 **BbITS detected in mouse blood and tissues at 1, 2 & 3 weeks post-inoculation.** Mixed BbITS infections were initiated in 9 mice by injecting a combined inoculum of 3.5×10^4^ organisms, with each of 7 tagged clones (A-G) present at an infectious dose of 5×103 spirochetes. Spirochete isolation was attempted with 150 µl of blood from each mouse at 1 week post-inoculation, a 2 mm ear punch biopsy of each mouse at 2 weeks post-inoculation, and ear, bladder, rear ankle joints and inoculation site of each mouse following euthanasia at 3 weeks post-inoculation. *B. burgdorferi* genomic DNA prepared from these outgrowth cultures was used in PCR screens to identify the BbITS present in tissues of individual mice at these times points. Each tagged clone (A-G) is graphically depicted as a different color and the presence of a color block denotes detection of this clone in the indicated sample, whereas no color block indicates the corresponding tagged clone was not present. No spirochetes were isolated from the blood sample of mouse #1, and the outgrowth culture from the ear of mouse #8 was contaminated and not analyzed (X).
(DOCX)

Figure S3 **BbITS detected in mouse blood and tissues at 1, 2 & 6 weeks post-inoculation.** Mixed BbITS infections were initiated in 9 mice by injecting a combined inoculum of 3.5×10^4^ organisms, with each of 7 tagged clones (A-G) present at an infectious dose of 5×103 spirochetes. Spirochete isolation was attempted with 150 µl of blood from each mouse at 1 week post-inoculation, a 2 mm ear punch biopsy of each mouse at 2 weeks post-inoculation, and ear, bladder, rear ankle joints and inoculation site of each mouse following euthanasia at 6 weeks post-inoculation. *B. burgdorferi* genomic DNA prepared from these outgrowth cultures was used in PCR screens to identify the BbITS present in tissues of individual mice at these times points. Each tagged clone (A-G) is graphically depicted as a different color and the presence of a color block denotes detection of this clone in the indicated sample, whereas no color block indicates the corresponding tagged clone was not present. X indicates that the culture was contaminated and not analyzed.
(DOCX)

Figure S4 **BbITS detected in mouse blood and tissues at 1, 2 & 11 weeks post-inoculation.** Mixed BbITS infections were initiated in 9 mice by injecting a combined inoculum of 3.5×10^4^ organisms, with each of 7 tagged clones (A-G) present at an infectious dose of 5×103 spirochetes. Spirochete isolation was attempted with 150 µl of blood from each mouse at 1 week post-inoculation, a 2 mm ear punch biopsy of each mouse at 2 weeks post-inoculation, and ear, bladder, rear ankle joints and inoculation site of each mouse following euthanasia at 11 weeks post-inoculation. *B. burgdorferi* genomic DNA prepared from these outgrowth cultures was used in PCR screens to identify the BbITS present in tissues of individual mice at these times points. Each tagged clone (A-G) is graphically depicted as a different color and the presence of a color block denotes detection of this clone in the indicated sample, whereas no color block indicates the corresponding tagged clone was not present. X indicates that the culture was contaminated and not analyzed.
(DOCX)

Figure S5 **BbITS detected in mouse blood and tissues at 1, 2 & 17 weeks post-inoculation.** Mixed BbITS infections were initiated in 9 mice by injecting a combined inoculum of 3.5×10^4^ organisms, with each of 7 tagged clones (A-G) present at an infectious dose of 5×103 spirochetes. Spirochete isolation was attempted with 150 µl of blood from each mouse at 1 week post-inoculation, a 2 mm ear punch biopsy of each mouse at 2 weeks post-inoculation, and ear, bladder, rear ankle joints and inoculation site of each mouse following euthanasia at 17 weeks post-inoculation. *B. burgdorferi* genomic DNA prepared from these outgrowth cultures was used in PCR screens to identify the BbITS present in tissues of individual mice at these times points. Each tagged clone (A-G) is graphically depicted as a different color and the presence of a color block denotes detection of this clone in the indicated sample, whereas no color block indicates the corresponding tagged clone was not present. X indicates that the culture was contaminated and not analyzed.
(DOCX)

Table S1 **Oligonucleotides.**
(DOCX)
