# Correction: Population Bottlenecks during the Infectious Cycle of the Lyme Disease Spirochete *Borrelia burgdorferi*

**DOI:** 10.1371/journal.pone.0108612

**Published:** 2014-09-17

**Authors:** The PLOS ONE Staff

The first author’s current address is not indicated. The current address for Ryan O. M. Rego is Biology Centre, Institute of Parasitology, Academy of Sciences of the Czech Republic, Ceske Budejovice, Czech Republic.
