# Supplementary figures and images for: Population Bottlenecks during the Infectious Cycle of the Lyme Disease Spirochete Borrelia burgdorferi
Source: PLoS One. 2014 Jun 30;9(6):e101009. doi: 10.1371/journal.pone.0101009 (PMC4076273; doi:10.1371/journal.pone.0101009)

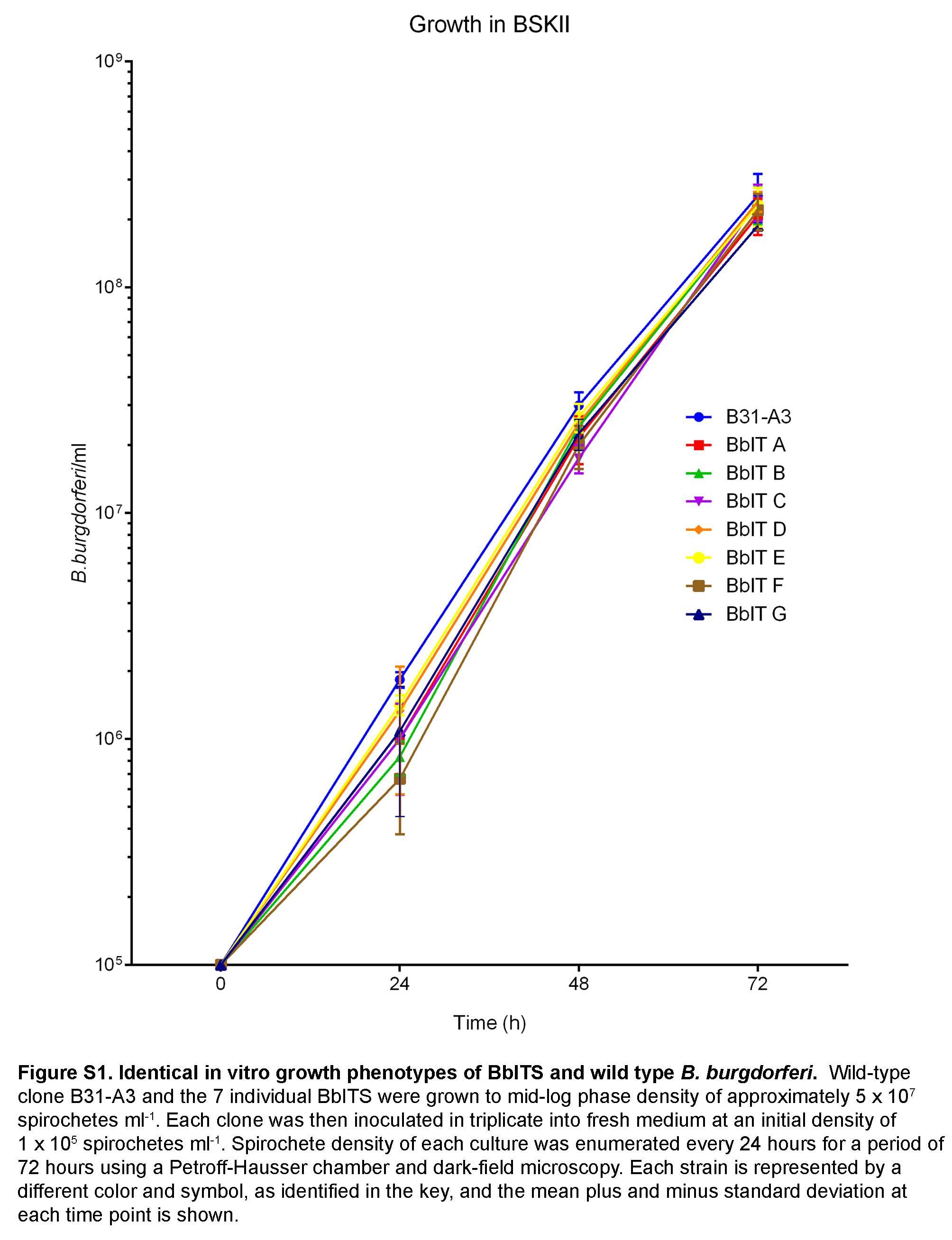

Supplement: Figure S1 — Identical in vitro growth phenotypes of BbITS and wild type B. burgdorferi . Wild-type clone B31-A3 and the 7 individual BbITS were grown to mid-log phase density of approximately 5×10^7^ spirochetes ml−1. Each clone was then inoculated in triplicate into fresh medium at an initial density of 1×10^5^ spirochetes ml−1. Spirochete density of each culture was enumerated every 24 hours for a period of 72 hours using a Petroff-Hausser chamber and dark-field microscopy. Each strain is represented by a different color and symbol, as identified in the key, and error bars indicate standard deviation from the mean. (TIF) [file pone.0101009.s001.tif]
