# Supplementary material for: Population Bottlenecks during the Infectious Cycle of the Lyme Disease Spirochete Borrelia burgdorferi
Source: PLoS One. 2014 Jun 30;9(6):e101009. doi: 10.1371/journal.pone.0101009 (PMC4076273; doi:10.1371/journal.pone.0101009)
Supplement: Figure S3 — BbITS detected in mouse blood and tissues at 1, 2 & 6 weeks post-inoculation. Mixed BbITS infections were initiated in 9 mice by injecting a combined inoculum of 3.5×10^4^ organisms, with each of 7 tagged clones (A-G) present at an infectious dose of 5×10^3^ spirochetes. Spirochete isolation was attempted with 150 µl of blood from each mouse at 1 week post-inoculation, a 2 mm ear punch biopsy of each mouse at 2 weeks post-inoculation, and ear, bladder, rear ankle joints and inoculation site of each mouse following euthanasia at 6 weeks post-inoculation. B. burgdorferi genomic DNA prepared from these outgrowth cultures was used in PCR screens to identify the BbITS present in tissues of individual mice at these times points. Each tagged clone (A-G) is graphically depicted as a different color and the presence of a color block denotes detection of this clone in the indicated sample, whereas no color block indicates the corresponding tagged clone was not present. X indicates that the culture was contaminated and not analyzed. (DOCX) [file pone.0101009.s003.docx]

**Figure S3**

| **No. 10** | **BbIT A** | **BbIT B** | **BbIT C** | **BbIT D** | **BbIT E** | **BbIT F** | **BbIT G** |
| --- | --- | --- | --- | --- | --- | --- | --- |
| **Blood** |  |  |  |  |  |  |  |
| **Ear biopsy** | ________ | ________ | ________ | ________ | ________ | ________ | _______ |
| **Ear** | ________ |  | ________ | ________ | ________ | ________ | _______ |
| **Bladder** | ________ | ________ | ________ | ________ | ________ | ________ | _______ |
| **Joint 1** | ________ | ________ | ________ | ________ | ________ | ________ | _______ |
| **Joint 2** | ________ | ________ | ________ |  | ________ | ________ | _______ |
| **Inoc. site** | ________ | ________ | ________ | ________ | ________ | ________ | _______ |

| **No. 11** | **BbIT A** | **BbIT B** | **BbIT C** | **BbIT D** | **BbIT E** | **BbIT F** | **BbIT G** |
| --- | --- | --- | --- | --- | --- | --- | --- |
| **Blood** |  |  | ________ |  |  |  | _______ |
| **Ear biopsy** | ________ |  | ________ | ________ | ________ | ________ | _______ |
| **Ear** | ________ |  | ________ | ________ | ________ | ________ | _______ |
| **Bladder** | ________ |  | ________ | ________ | ________ | ________ | _______ |
| **Joint 1** |  |  | ________ | ________ | ________ | ________ | _______ |
| **Joint 2** | ________ |  | ________ | ________ | ________ | ________ | _______ |
| **Inoc. site** | X | X | X | X | X | X | X |

| **No. 12** | **BbIT A** | **BbIT B** | **BbIT C** | **BbIT D** | **BbIT E** | **BbIT F** | **BbIT G** |
| --- | --- | --- | --- | --- | --- | --- | --- |
| **Blood** |  |  | ________ | ________ |  |  | _______ |
| **Ear biopsy** | ________ | ________ | ________ | ________ | ________ | ________ | _______ |
| **Ear** | ________ | ________ | ________ | ________ | ________ | ________ | _______ |
| **Bladder** | ________ |  |  |  | ________ | ________ | _______ |
| **Joint 1** |  |  |  |  |  |  | _______ |
| **Joint 2** |  |  |  |  |  |  | _______ |
| **Inoc. site** | ________ |  |  |  |  |  | _______ |

| **No. 13** | **BbIT A** | **BbIT B** | **BbIT C** | **BbIT D** | **BbIT E** | **BbIT F** | **BbIT G** |
| --- | --- | --- | --- | --- | --- | --- | --- |
| **Blood** |  |  | ________ | ________ |  |  | _______ |
| **Ear biopsy** | ________ | ________ | ________ |  | ________ | ________ | _______ |
| **Ear** | ________ |  | ________ |  | ________ | ________ | _______ |
| **Bladder** | ________ |  | ________ |  | ________ | ________ | _______ |
| **Joint 1** | ________ | ________ | ________ | ________ | ________ | ________ | _______ |
| **Joint 2** | ________ | ________ | ________ |  | ________ | ________ | _______ |
| **Inoc. site** | ________ | ________ | ________ | ________ | ________ | ________ | _______ |

| **No. 14** | **BbIT A** | **BbIT B** | **BbIT C** | **BbIT D** | **BbIT E** | **BbIT F** | **BbIT G** |
| --- | --- | --- | --- | --- | --- | --- | --- |
| **Blood** |  |  |  |  |  |  |  |
| **Ear biopsy** | ________ | ________ | ________ | ________ | ________ | ________ | _______ |
| **Ear** | ________ | ________ | ________ | ________ | ________ |  | _______ |
| **Bladder** |  | ________ | ________ | ________ | ________ | ________ | _______ |
| **Joint 1** |  | ________ | ________ | ________ | ________ | ________ |  |
| **Joint 2** |  |  |  | ________ |  | ________ | _______ |
| **Inoc. site** | ________ | ________ | ________ | ________ | ________ |  | _______ |

| **No. 15** | **BbIT A** | **BbIT B** | **BbIT C** | **BbIT D** | **BbIT E** | **BbIT F** | **BbIT G** |
| --- | --- | --- | --- | --- | --- | --- | --- |
| **Blood** |  |  | ________ |  |  | ________ |  |
| **Ear biopsy** | ________ | ________ | ________ | ________ | ________ | ________ | _______ |
| **Ear** | ________ | ________ | ________ | ________ | ________ | ________ | _______ |
| **Bladder** | ________ | ________ | ________ |  |  | ________ | _______ |
| **Joint 1** | ________ | ________ | ________ | ________ |  | ________ | _______ |
| **Joint 2** |  |  | ________ |  |  |  | _______ |
| **Inoc. site** | ________ | ________ | ________ | ________ |  |  |  |

| **No. 16** | **BbIT A** | **BbIT B** | **BbIT C** | **BbIT D** | **BbIT E** | **BbIT F** | **BbIT G** |
| --- | --- | --- | --- | --- | --- | --- | --- |
| **Blood** | ________ |  | ________ |  | ________ |  | _______ |
| **Ear biopsy** | ________ |  | ________ | ________ | ________ |  | _______ |
| **Ear** | ________ |  | ________ | ________ | ________ |  |  |
| **Bladder** |  |  | ________ | ________ | ________ |  | _______ |
| **Joint 1** |  |  |  |  | ________ |  |  |
| **Joint 2** |  |  |  |  | ________ |  | _______ |
| **Inoc. site** | ________ |  | ________ | ________ | ________ |  | _______ |

| **No. 17** | **BbIT A** | **BbIT B** | **BbIT C** | **BbIT D** | **BbIT E** | **BbIT F** | **BbIT G** |
| --- | --- | --- | --- | --- | --- | --- | --- |
| **Blood** |  |  |  |  |  |  |  |
| **Ear biopsy** | ________ |  | ________ | ________ | ________ | ________ | _______ |
| **Ear** | ________ |  | ________ | ________ | ________ | ________ | _______ |
| **Bladder** | ________ |  | ________ | ________ | ________ | ________ |  |
| **Joint 1** | X | X | X | X | X | X | X |
| **Joint 2** | X | X | X | X | X | X | X |
| **Inoc. site** | ________ | ________ | ________ | ________ | ________ | ________ | _______ |

| **No. 18** | **BbIT A** | **BbIT B** | **BbIT C** | **BbIT D** | **BbIT E** | **BbIT F** | **BbIT G** |
| --- | --- | --- | --- | --- | --- | --- | --- |
| **Blood** |  |  | ________ | ________ |  |  |  |
| **Ear biopsy** | ________ | ________ | ________ | ________ | ________ | ________ | _______ |
| **Ear** | ________ |  | ________ | ________ | ________ | ________ | _______ |
| **Bladder** | ________ |  | ________ | ________ | ________ | ________ | _______ |
| **Joint 1** | ________ |  | ________ |  | ________ | ________ | _______ |
| **Joint 2** | ________ | ________ | ________ | ________ | ________ | ________ | _______ |
| **Inoc. site** | ________ | ________ | ________ | ________ | ________ | ________ | _______ |
