# Supplementary material for: Population Bottlenecks during the Infectious Cycle of the Lyme Disease Spirochete Borrelia burgdorferi
Source: PLoS One. 2014 Jun 30;9(6):e101009. doi: 10.1371/journal.pone.0101009 (PMC4076273; doi:10.1371/journal.pone.0101009)
Supplement: Table S1 — Oligonucleotides. (DOCX) [file pone.0101009.s006.docx]

**Table S1 Oligonucleotides**

| **BbITS construction & screening^a^** | **Sequence**^b^ |
| --- | --- |
| tag A-f | agtact**CGCGCGATAGGTGTGGAGTG**TAATACCCGAGCTTCAAGGAAGA |
| tag A-r | gtatac**AACAACCCCCCCAACGACAC**AAGCCGATCTCGGCTTGAACGA |
| tag B-f | agtact**GGCGAGGGCGCGAGTGAGAT**TAATACCCGAGCTTCAAGGAAGA |
| tag B-r | gtatac**ATATCAATCCCACACACTCG**AAGCCGATCTCGGCTTGAACGA |
| tag C-f | agtact**AGCTGGAGCTCGAGAGTGAG**TAATACCCGAGCTTCAAGGAAGA |
| tag C-r | gtatac**ATAGCTAAAGACACCCCTCA**AAGCCGATCTCGGCTTGAACGA |
| tag D-f | agtact**GGCGTGGGAGTGAGGGGGTG**TAATACCCGAGCTTCAAGGAAGA |
| tag D-r | gtatac**AACTATAACGCTCACCAGCC**AAGCCGATCTCGGCTTGAACGA |
| tag E-f | agtact**AGGGGTGGGGTTATGGGGCT**TAATACCCGAGCTTCAAGGAAGA |
| tag E-r | gtatac**AGCTAGCACGCGCACTATCC**AAGCCGATCTCGGCTTGAACGA |
| tag F-f | agtact**ATTGGGATGGCGGTATAGTT**TAATACCCGAGCTTCAAGGAAGA |
| tag F-r | gtatac**AAATCAAGAACGATATACCT**AAGCCGATCTCGGCTTGAACGA |
| tag G-f | agtact**AGAGAGAGCTGGAGAGTGAT**TAATACCCGAGCTTCAAGGAAGA |
| tag G-r | gtatac**CACTCCCACCACAACCACCG**AAGCCGATCTCGGCTTGAACGA |

| Gent-F | TCTCGGCTTGAACGAATTGTTACGT |
| --- | --- |
| Gent-R | GGCAGTCGCCCTAAAACAAAGTT |
| BBE_05F | ATTCATTTATCACTAGAGTTTG |
| BBE_06R | AAGGTGTCGTAATAGGGC |

^a^Oligonucleotide primers used in the generation of allelic exchange constructs (tags A-G) and subsequent screening of resulting *B. burgdorferi* clones (BbITS ).

^b^Restriction enzyme recognition sequences are denoted in lower case. Unique 20 base sequence tags introduced into each BbITS are shown in underlined bold font and also represent the primers used for subsequent identification of individual BbITS.
